# Impaired proteasome activity and neurodegeneration with brain iron accumulation in *FBXO7* defect

**DOI:** 10.1002/acn3.51095

**Published:** 2020-08-06

**Authors:** Marta Correa‐Vela, Vincenzo Lupo, Marta Montpeyó, Paula Sancho, Anna Marcé‐Grau, Jorge Hernández‐Vara, Alejandra Darling, Alison Jenkins, Sandra Fernández‐Rodríguez, Cristina Tello, Laura Ramírez‐Jiménez, Belén Pérez, Ángel Sánchez‐Montáñez, Alfons Macaya, María J. Sobrido, Marta Martinez‐Vicente, Belén Pérez‐Dueñas, Carmen Espinós

**Affiliations:** ^1^ Department of Pediatric Neurology Hospital Universitari Vall d’Hebron, Vall d´Hebron Institut de Recerca Barcelona Spain; ^2^ Universitat Autònoma de Barcelona Barcelona Spain; ^3^ Unit of Genetics and Genomics of Neuromuscular and Neurodegenerative Disorders Centro de Investigación Príncipe Felipe (CIPF) Valencia Spain; ^4^ Joint Units INCLIVA & IIS La Fe Rare Diseases Centro de Investigación Príncipe Felipe (CIPF) Valencia Spain; ^5^ Neurodegenerative diseases‐CIBERNED Vall d´Hebron Institut de Recerca Barcelona Spain; ^6^ Department of Neurology Hospital Universitari Vall d’Hebron Barcelona Spain; ^7^ Department of Pediatric Neurology Hospital Sant Joan de Déu Barcelona Spain; ^8^ Unit of Genomics and Traslational Genetics Centro de Investigación Príncipe Felipe (CIPF) Valencia Spain; ^9^ Department of Molecular Biology Centro de Biología Molecular Severo‐Ochoa UAM‐CSIC Universidad Autónoma de Madrid Centro de Diagnóstico de Enfermedades Moleculares (CEDEM) CIBER on Rare Diseases (CIBERER) Instituto de Investigación Sanitaria Hospital La Paz (IdiPaz) Madrid Spain; ^10^ Department of Pediatric Radiology Hospital Universitari Vall d’Hebrón Barcelona Spain; ^11^ Neurogenetics Research Group Instituto de Investigaciones Sanitarias (IDIS) Fundación Pública Galega de Medicina Xenómica, and CIBER on Rare Diseases (CIBERER) Santiago de Compostela Spain

## Abstract

FBXO7 is implicated in the ubiquitin–proteasome system and parkin‐mediated mitophagy. FBXO7defects cause a levodopa‐responsive parkinsonian‐pyramidal syndrome(PPS). Methods: We investigated the disease molecular bases in a child with PPS and brain iron accumulation. Results: A novel homozygous c.368C>G (p.S123*) FBXO7 mutation was identified in a child with spastic paraplegia, epilepsy, cerebellar degeneration, levodopa nonresponsive parkinsonism, and brain iron deposition. Patient’s fibroblasts assays demonstrated an absence of FBXO7 RNA expression leading to impaired proteasome degradation and accumulation of poly‐ubiquitinated proteins. Conclusion: This novel FBXO7 phenotype associated with impaired proteasome activity overlaps with neurodegeneration with brain iron accumulation disorders.

## Introduction

Parkinsonism‐pyramidal syndrome (PPS) is a complex movement disorder that may present in childhood associated with complex hereditary spastic paraplegia (SPG), young‐onset parkinsonism, neurodegeneration with brain iron accumulation (NBIA) disorders, and inborn errors of metabolism.^1^ FBXO7 deficiency (PARK15; MIM 260300) is a rare cause of PPS with 26 known patients since 2008.^2^, ^3^, ^4^, ^5^, ^6^, ^7^, ^8^, ^9^ Most of these patients develop spastic paraparesia and/or levodopa‐responsive young onset parkinsonism with normal brain magnetic resonance imaging (MRI) and reduced dopamine uptake on DaTSCAN.^2^, ^3^, ^4^, ^5^, ^6^, ^7^, ^8^, ^9^ The F‐box only protein7 (*FBXO7)* gene encodes for a protein involved in ubiquitin–proteasome system (UPS) activity and mitophagy. FBXO7 protein is a subunit of a complex responsible for the phosphorylation‐dependent ubiquitination, the SKP1/cullin‐1/F‐box protein (SCF) E3 ubiquitin ligase complex. FBXO7 acts as a substrate recruiter in this complex and has a regulatory role in proteasome through the direct interaction with the core proteasome subunit PSMA2, an essential protein required for a fully functional proteasome. Another role of FBXO7 protein is to provide an effective recruitment of parkin to damaged mitochondria and initiate mitophagy.^10^, ^11^, ^12^, ^13^

The genetic analysis of a child with a complex phenotype including epilepsy, progressive PPS with no response to levodopa, cognitive decline, and MRI features suggestive of NBIA revealed the novel homozygous *FBXO7* c.368C>G (p.S123*) variant. We detected for the first time in patient’s fibroblasts an impairment in ubiquitin‐protein degradation and poly‐ubiquitinated (poly‐UB) proteins accumulation, (highlighting the essential role of the UPS activity of FBXO7 in human disease.

## Methods

Used methodology is summarized in the main manuscript. Complete procedures and protocols are detailed in Supporting Information.

### Consent and approval

All protocols complied with the ethics guidelines according to the Declaration of Helsinki and were approved by participating institutional review boards. The proband and relatives signed an informed consent for the study.

### Genetic studies

Genetic test was performed in the proband using a customized gene panel. Because of second‐grade parental consanguinity, homozygous variants were prioritized.

### Cell culture and western‐blot

Skin cultured fibroblasts from the patient and controls were grown in standard medium. FBXO7 expression and mitophagy‐related proteins (PINK1, Parkin, K63 poly‐UB, VDAC1) were studied by Western blot in homogenate and mitochondrial enriched fractions.

### Nonsense‐mediated mRNA decay (NMD) assay

Proband and control fibroblasts cultures were treated with emetine dihydrochloride hydrate,^14^ a specific inhibitor of translation. RNA was extracted and retro‐transcribed, and the resulting cDNAs underwent qPCR for the quantification of *FBXO7* RNA expression.

### Ubiquitin–proteasome system activity and expression assays

Chymotrypsin‐like activity determination was done measuring fluorescence intensity after incubation of the proband and control fibroblasts’ protein extract with a proteasome activity buffer and a fluorogenic substrate. Ubiquitinated‐protein levels were quantified through a WB assay using an anti‐UB antibody (Sigma‐Aldrich, St. Louis, USA).

### Electronic microscopy

Ultra‐thin sections were examined under a transmission electron microscope FEI Tecnai Spirit BioTwin using a digital camera Morada (see Supplemental File).

## Results

### Case Presentation

The proband is currently a 21‐year‐old girl born to healthy consanguineous parents from Morocco. After a normal perinatal history, she presented with mild global developmental delay followed by acute ataxia triggered by a viral gastroenteritis at 2 years. When she was 5 years old, valproate was started for absence epilepsy. She was lost to follow‐up until the age of 12, when she was admitted to the intensive care unit due to acute parainfectious H1N1 encephalopathy. Neurological examination identified horizontal nystagmus, head tremor, dysmetria, lower limb spasticity, hyperreflexia, clonus, bilateral Babinski sign, and bladder dysfunction.

Between the age of 12 and 18 years the patient suffered progressive deterioration, including progressive spastic paraparesia and gait loss at 13 years, language and cognitive regression, drug‐resistant seizures, severe dysphagia, and optic neuropathy. At the age of 15, hypokinesia, rigidity, kinetic tremor, and upgaze limitation were identified. A trial with levodopa (100g/day) did not show a positive response (Video).

**Figure 1:**
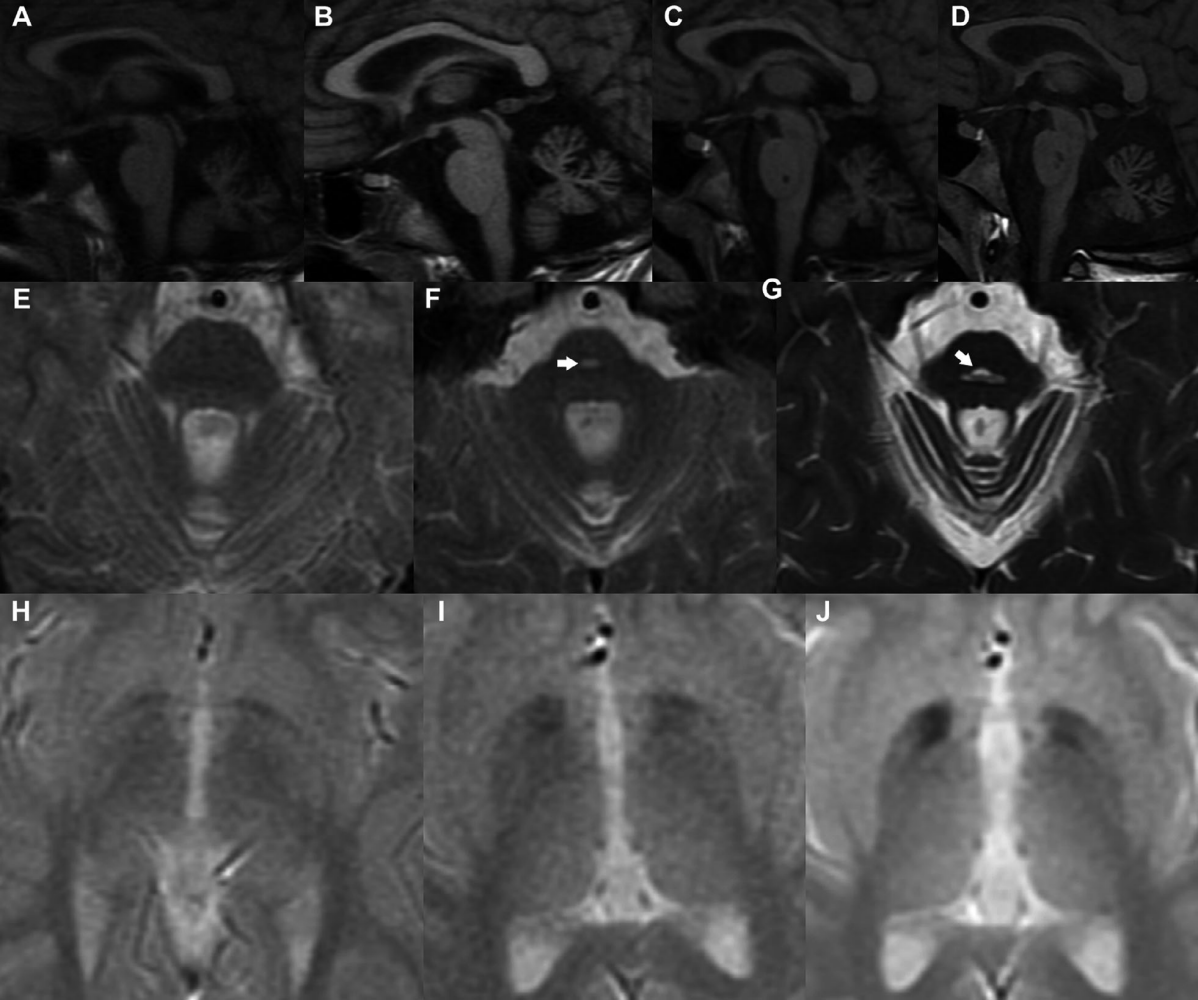
MRI proband’s studies: T1WI sagittal images (A) at 10 years old (B), 12 years old (C), 15 years old and (D) 17 years old show a progression from slight vermian atrophy to cerebellar atrophy of vermis and hemispheres, cerebral volume loss and brainstem atrophy, enlarged ventricles, and cerebral sulci at the age of 17. Axial T2WI of the pons at (E) 12 years old, (F) 15 years old and (G) 17 years old. T2 hyperintensity delineating a cross through the pons known as the “hot cross bun” sign and representing selective degeneration of pontocerebellar tracts appear at the age of 17. Gradient recall echo [GRE] sequence basal ganglia at (H) 12 years old (I), 15 years old and (J) and 17 years. Bilateral hypointensities in pallidum suggest that iron deposition appeared at 15 years.

Serial brain and medulla MRI studies from 10 to 17 years disclosed slight vermian atrophy progressing to cerebral and cerebellar atrophy, and a hot cross bun sign suggesting ponto‐cerebellar tract degeneration. Excessive brain iron deposition in the pallidum and substantia nigra appeared as hypointense signal on iron‐sensitive sequences at 15 years, which led us to suspect a causative relation with the onset of parkinsonian features (Fig. 1). At 20 years, a brain DaTSCAN showed normal uptake.

### Genetic findings

**Figure 2:**
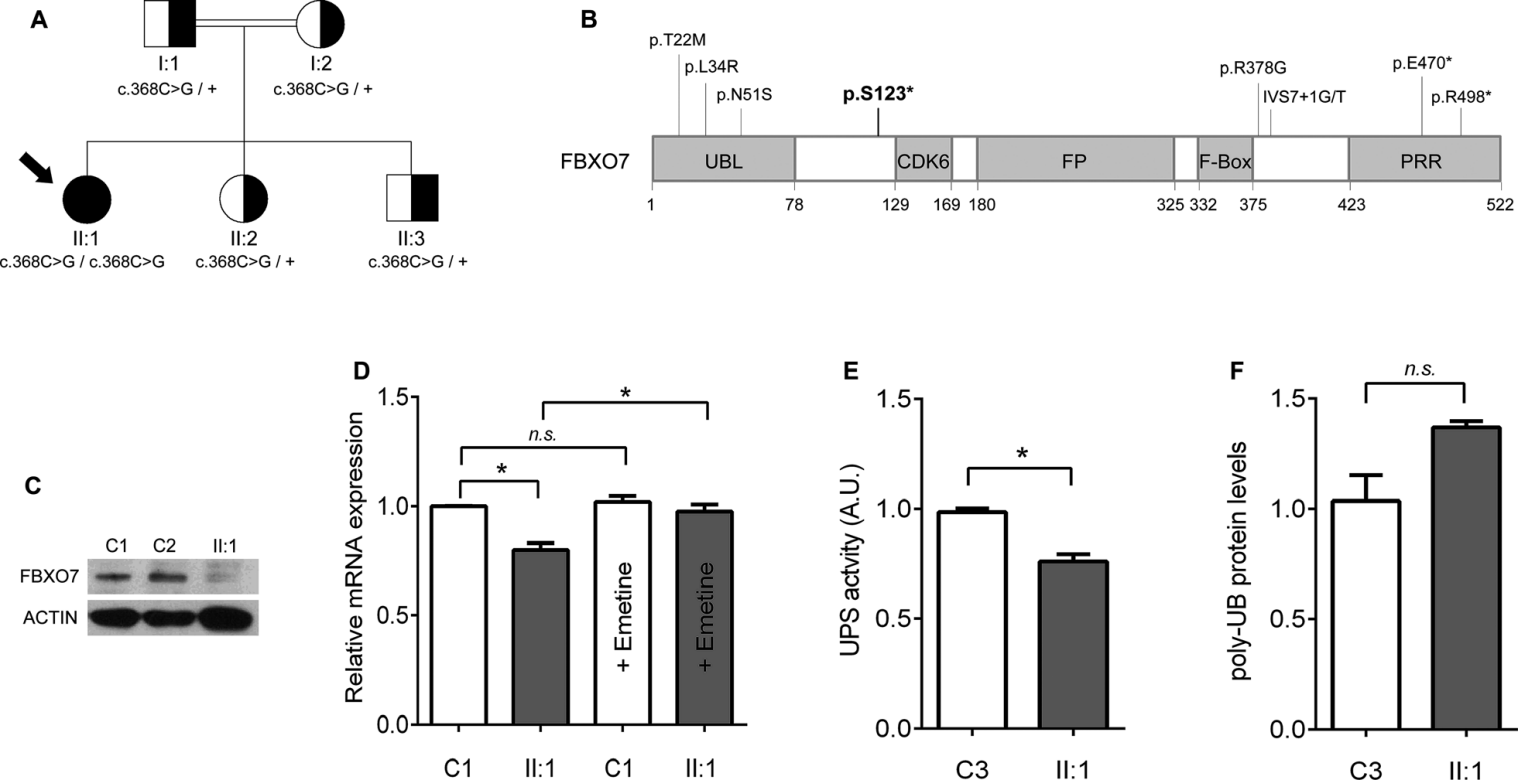
Pedigree and analysis of the FBXO7 p.S123* mutation: (A) FBXO7 c.368C>G (p.S123*) mutation family’s pedigree. The index case (II:1) is homozygous for the mutation (black circle with an arrow). Her healthy relatives are heterozygous carriers (half‐shaded symbols). (B) Distribution of pathogenic mutations reported in FBXO7 according to protein domains; p.S123* (in bold) is located in an inter‐domain between the UBL and the CDK6 domains. (C) Absence of FBXO7 protein western blot in patient’s fibroblasts (II:1) compared to two controls fibroblasts’ lysates (C1 and C2) immunodetected with an antibody against FBXO7. (D) mRNA expression was evaluated by qPCR in fibroblasts from the patient (II:1) and one control with and without emetine treatment, a translation inhibitor. FBXO7 mRNA levels in patient’s fibroblasts (II:1) are rescued when treated with emetine. Data were obtained from one biological sample and three independent technical replicates. Error bars represent SEM. Mann–Whitney–Wilcoxon test: *P < 0.05; n.s: nonsignificant. (E) UPS activity assay performed in fibroblasts from the patient (II:1) and one control (C3) shows a reduction of 20% UPS activity in patient. Data were obtained from one biological sample and six independent technical replicates. Error bars represent SEM. Mann–Whitney–Wilcoxon test: *P < 0.05. (F) Detection of a trend to increase in poly‐UB proteins in patient (II:1) fibroblasts by Western blot and SDS‐PAGE. Actin was used to validate equal protein loading. Data were obtained from one biological sample and three independent technical replicates. Error bars represent SEM. Mann–Whitney–Wilcoxon test. n.s: nonsignificant

Data analysis of a custom gene panel identified two homozygous variants: c.100A>G (p.T34A) in *DLD* (NM_000108.4) and c.368C>G (p.S123*) in *FBXO7* (NM_012179.3) (Fig. 2A). In parallel, exome sequencing analysis revealed the same findings (data not shown). *In silico* predictors showed that DLD p.T34A (rs138002793; MAF = 5.1 × 10^‐4^) was probably benign. DLD enzyme activity assay in patient's fibroblasts was considered within the normal range of activity. According to these findings and the patient’s clinical record, we ruled out the implication of *DLD*. Although gene panel data analysis disclosed a moderate number of single‐exon CNVs in the proband, array‐CGH found no pathological results (data not shown).

### Functional studies

The novel *FBXO7* c.368C>G (p.S123*) change would produce a very short protein where the only functional domain preserved would be the ubiquitin‐like domain (Fig. 2B). Protein expression analysis showed the absence of FBXO7 protein in WB (Fig. 2C). mRNA expression was investigated by NMD assay confirming a significant decrease in *FBXO7* mRNA in patient’s fibroblasts compared to control (*P* < 0.05). Emetine incubation increased total mRNA in patient’s fibroblasts up to control levels, thus confirming a NMD mechanism as a cause of loss of function effect (Fig. 2D).

Loss of FBXO7 is related to decreased proteasome activity.^10^ A significant reduction of 20% in UPS activity was observed in patient’s fibroblasts (Fig. 2E). We hypothesized that impaired UPS would not be able to degrade the poly‐UB labeled proteins and we found an increase in these proteins in proband’s fibroblasts (Fig. 2F).

FBOX7 is also involved in mitophagy.^12^, ^13^ Mitophagy‐related proteins studies showed no significant differences in mitochondrial enriched fractions in patient’s fibroblasts (Fig. S1A). However, elevated mitochondrial proteins in mutant cells were observed, possibly due to accumulated mitochondria (Fig. S1B). Mitochondria analysis by electron microscopy revealed abnormal cristae morphology in ultra‐thin sections images (Fig. S1C).

## Discussion

FBXO7 deficiency is a rare genetic condition described in 26 patients from 10 unrelated families, giving three major phenotypes: autosomal recessive juvenile‐onset levodopa responsive PPS (14 patients), SPG (7 patients), and isolated parkinsonism (5 patients).^2^, ^3^, ^4^, ^5^, ^6^, ^7^, ^8^, ^9^ We report a patient with a novel, complex, and severe form of childhood onset FBXO7*‐*PPS with initial developmental delay and progressive SPG, drug‐resistant epilepsy, levodopa nonresponsive parkinsonism, and signs of cerebellar degeneration and basal ganglia iron deposition.

We observed a striking association between the onset of parkinsonian features and the occurrence of brain iron accumulation on MRI at 15 years. These findings, together with poor levodopa response and normal dopamine uptake suggested a likely postsynaptic degeneration condition overlapping with NBIA disorders. This contrasts previous reports showing a severe nigrostriatal dopaminergic defect in two patients carrying *FBXO7* mutations.^3^, ^4^

Moreover, the patient suffered from cerebellar ataxia, oculomotor dysfunction, severe dysphagia, and cognitive decline. These features could be related to the progression of cerebellar atrophy and the appearance of a hot cross bun sign at the pons, also described in patients with multiple system atrophy or spinocerebellar ataxia.^15^ Neither brain iron accumulation nor the hot cross bun sign have been reported in *FBXO7* patients, mostly having normal structure or mild global brain atrophy on MRI (Table S1).

Previous *FBXO7* patients with nonsense variants had the onset of core features within the second decade of life, and progression to severe parkinsonism, major disability or early death in five of 13 cases.^3^, ^4^, ^5^, ^6^, ^8^, ^9^ However, none of them developed epilepsy or cerebellar ataxia, two atypical features of our patient. On the other hand, patients with missense variants showed milder phenotypes, such as young onset parkinsonism or isolated SPG, probably due to a possible residual activity of FBXO7^7^ (Table. S1).

The *FBXO7* c.368C>G (p.S123*) nonsense mutation is located in the proximal region of the protein. According to our results, it would produce an aberrant mRNA, which was degraded by NMD, leading to a complete absence of FBXO7 protein and a loss of function effect. NMD is a well‐known RNA quality control that limits the expression of mRNAs with premature termination codons.^16^, ^17^ Previously, two additional nonsense *FBXO7* mutations (p.R498* and p.E470*) were reported.^3^, ^9^ The absence of expression of p.R498* was demonstrated in a human cell model, however, NMD process was discarded probably due to its location near the C‐ter.^18^

FBXO7 plays a regulatory role in proteasome and mitochondria. The *FBXO7* knockdown in mice results in a decrease in UPS activity.^10^ We have demonstrated that the UPS activity was reduced in FBXO7‐PPS patient’s fibroblasts, and as a consequence, the poly‐UB proteins showed a trend to accumulate. Moreover, our findings suggest a likely mechanistic relationship between proteasome impairment and iron accumulation, previously observed in experimental models of PD. A mice model of PD induced by the proteasome inhibitor lactacystin showed loss of dopamine neurons, abnormal iron accumulation, and elevated iron regulatory protein 2 (IRP2) and divalent metal transporter 1 (DMT1) in the substantia nigra.^19^ Also, the in vitro lactacystin dopamine cell line model showed an increase in labile iron, disruption in iron regulatory proteins, and aggregation of ubiquitin‐conjugated proteins, leading to cell premature death.^20^ Taken together, these findings suggest that alterations in iron regulatory proteins due to proteasome dysfunction may lead to dopamine neuron degeneration.

In addition, PINK1/Parkin‐mediated mitophagy is facilitated by FBXO7 and dependent on ubiquitin.^11^, ^13^, ^21^, ^22^, ^23^ We have not found differences in the expression of mitophagy‐related proteins between mutant and healthy fibroblasts. However, elevated mitochondrial proteins in mutant cells were observed, possibly due to accumulated mitochondria.

Mitochondrial cristae alterations are associated with mitochondria impairment.^24^ In a transgenic *Drosophila* model, the overexpression of *FBXO7* leads to broken cristae and accumulated high‐density material in the swollen mitochondria.^11^ We observed abnormal mitochondrial cristae morphology in patient’s fibroblasts, likely as a secondary consequence, since it is commonly seen in neurodegenerative diseases, including NBIA forms.^25^, ^26^, ^27^

Our results have major limitations and should be interpreted with caution as they have been demonstrated in a single patient harbouring FBXO7 p.S123* mutation. Further studies are needed to clarify if proteosome impairment, mitophagy dysfunction, and iron accumulation are the main biological mechanisms leading to PPS in FBXO7 patients.

In conclusion, we describe a complex and severe FBXO7‐related phenotype with a likely postsynaptic mechanism of neurodegeneration, which overlaps with other NBIA monogenic diseases. The absence of *FBXO7* led to impaired proteasome degradation and accumulation of poly‐ubiquitinated proteins, which highlights the essential role of the UPS in the disease mechanism.

## Authors' contributions

Research project: A. Conception, B. Organization, C. Execution: VL, MCV, AD, JHV, BPD, CE. Experimental studies and statistical analysis: A. Design, B. Execution: PS, VL, MM, AMG, SFR, AJ, CT, LR, BP, MJS, MMV. C. Review and critique MCV, VL, MMV, BPD, CE. Manuscript preparation: A. Writing of the first draft, B. Review and critique MCV, VL, AMG, MMV, BPD, CE. All the authors have reviewed and criticized the manuscript.

## Conflict of interests

Authors report no disclosures.

## Supporting information


**Video Segment A** **.** The patient at 13 years old is in a wheelchair due to spastic paraplegia.
**Video Segment B** **.** The patient at 15 years old has severe hypomimia, bradykinesia, and low amplitude kinetic tremor while throwing the ball.
**Video Segment C** **.** Oculomotor features of patient at 16 years old are horizontal nystagmus and reduced upgaze during smooth pursuit.
**Video Segment D** **.** Neurologic assessment at 21 years old reveals lower limbs hyperreflexia, bilateral Babinski sign, and Achilles clonus. On a second frame, we also observe upper limb bradykinesia, action tremor, and myoclonus.

**File S1** **.** Additional information regarding materials and methods, a supplementary figure and a supplementary table are included in a supplementary material file.
**Figure S1** **.** Mitophagy‐related proteins and electron microscopy: (A) Mitophagy markers (PINK1, Parkin, K63‐polyUb, and VDAC) levels analyzed by Western‐blot in mitochondria enriched fractions from patient's and control’s fibroblasts. Error bars represent SEM. n.s.: nonsignificant. (B) Elevated levels of mitochondrial‐specific proteins PINK1 and VDAC1 were detected in mutant cells compared to controls. Error bars represent SEM. Mann–Whitney–Wilcoxon test: *P < 0.05; n.s.: nonsignificant. (C) Analysis of mitochondrial morphology by electron microscopy revealed in ultrathin sections images of mitochondria in control’s and patient’s fibroblasts. Internal scales marker are indicated in the figure.
**Table S1** **.** Genetic, clinical, and neuroimaging features of FBXO7 deficiency
**Supplementary Material**

## References

[1] Tranchant C , Koob M , Anheim M . Parkinsonian‐Pyramidal syndromes: A systematic review. Parkinsonism Relat Disord 2017;39:4–16. 28256436 10.1016/j.parkreldis.2017.02.025

[2] Shojaee S , Sina F , Banihosseini SS , et al. Genome‐wide linkage analysis of a Parkinsonian‐pyramidal syndrome pedigree by 500 K SNP arrays. Am J Hum Genet 2008;82(6):1375–1384. 18513678 10.1016/j.ajhg.2008.05.005 PMC2427312

[3] Di Fonzo A , Dekker MC , Montagna P , et al. FBXO7 mutations cause autosomal recessive, early‐onset parkinsonian‐pyramidal syndrome. Neurology 2009;72(3):240–245. 19038853 10.1212/01.wnl.0000338144.10967.2b

[4] Paisan‐Ruiz C , Guevara R , Federoff M , et al. Early‐onset L‐dopa‐responsive parkinsonism with pyramidal signs due to ATP13A2, PLA2G6, FBXO7 and spatacsin mutations. Mov Disord 2010;25(12):1791–1800. 20669327 10.1002/mds.23221 PMC6005705

[5] Yalcin‐Cakmakli G , Olgiati S , Quadri M , et al. A new Turkish family with homozygous FBXO7 truncating mutation and juvenile atypical parkinsonism. Parkinsonism Relat Disord 2014;20(11):1248–1252. 25085748 10.1016/j.parkreldis.2014.06.024

[6] Gunduz A , Eken AG , Bilgic B , et al. FBXO7‐R498X mutation: phenotypic variability from chorea to early onset parkinsonism within a family. Parkinsonism Relat Disord 2014;20(11):1253–1256. 25169713 10.1016/j.parkreldis.2014.07.016

[7] Lohmann E , Coquel AS , Honore A , et al. A new F‐box protein 7 gene mutation causing typical Parkinson's disease. Mov Disord 2015;30(8):1130–1133. 26010069 10.1002/mds.26266

[8] Conedera S , Apaydin H , Li Y , et al. FBXO7 mutations in Parkinson's disease and multiple system atrophy. Neurobiol Aging 2016;40(192):192.e1–192.e5. 10.1016/j.neurobiolaging.2016.01.003 26882974

[9] Wei L , Ding L , Li H , et al. Juvenile‐onset parkinsonism with pyramidal signs due to compound heterozygous mutations in the F‐Box only protein 7 gene. Parkinsonism Relat Disord 2018;47:76–79. 29174172 10.1016/j.parkreldis.2017.11.332

[10] Vingill S , Brockelt D , Lancelin C , et al. Loss of FBXO7 (PARK15) results in reduced proteasome activity and models a parkinsonism‐like phenotype in mice. The EMBO journal 2016;35(18):2008–2025. 27497298 10.15252/embj.201593585 PMC5282834

[11] Zhou ZD , Xie SP , Sathiyamoorthy S , et al. F‐box protein 7 mutations promote protein aggregation in mitochondria and inhibit mitophagy. Hum Mol Genet 2015;24(22):6314–6330. 26310625 10.1093/hmg/ddv340

[12] Zhou ZD , Lee JCT , Tan EK . Pathophysiological mechanisms linking F‐box only protein 7 (FBXO7) and Parkinson's disease (PD). Mutat Res 2018;778:72–78. 30454685 10.1016/j.mrrev.2018.10.001

[13] Burchell VS , Nelson DE , Sanchez‐Martinez A , et al. The Parkinson's disease‐linked proteins Fbxo7 and Parkin interact to mediate mitophagy. Nat Neurosci 2013;16(9):1257–1265. 23933751 10.1038/nn.3489 PMC3827746

[14] Kawarai T , Yamasaki K , Mori A , et al. MFN2 transcripts escaping from nonsense‐mediated mRNA decay pathway cause Charcot‐Marie‐Tooth disease type 2A2. J Neurol Neurosurg Psychiatry 2016;87(11):1263–1265. 27154191 10.1136/jnnp-2015-312646

[15] Lee YC , Liu CS , Wu HM , et al. The 'hot cross bun' sign in the patients with spinocerebellar ataxia. Eur J Neurol 2009;16(4):513–516. 19187260 10.1111/j.1468-1331.2008.02524.x

[16] Fatscher T , Boehm V , Gehring NH . Mechanism, factors, and physiological role of nonsense‐mediated mRNA decay. Cell Mol Life Sci 2015;72(23):4523–4544. 26283621 10.1007/s00018-015-2017-9 PMC11113733

[17] Miller JN , Pearce DA . Nonsense‐mediated decay in genetic disease: friend or foe? Mutat Res, Rev Mutat Res 2014;762:52–64. 25485595 10.1016/j.mrrev.2014.05.001 PMC4260155

[18] Zhao T , De Graaff E , Breedveld GJ , et al. Loss of nuclear activity of the FBXO7 protein in patients with Parkinsonian‐Pyramidal Syndrome (PARK15). PLoS One 2011;6(2):e16983. 21347293 10.1371/journal.pone.0016983 PMC3037939

[19] Xie W , Li X , Li C , et al. Proteasome inhibition modeling nigral neuron degeneration in Parkinson’s disease. J Neurochem 2010;115:188–199. 20649845 10.1111/j.1471-4159.2010.06914.x

[20] Li XP , Xie WJ , Zhang Z , et al. A mechanistic study of proteasome inhibition‐induced iron misregulation in dopamine neuron degeneration. Neurosignals 2012;20(4):223–236. 22269801 10.1159/000332954

[21] Scarffe LA , Stevens DA , Dawson VL , Dawson TM . Parkin and PINK1: much more than mitophagy. Trends Neurosci 2014;37(6):315–324. 24735649 10.1016/j.tins.2014.03.004 PMC4075431

[22] Whitworth AJ , Pallanck LJ . PINK1/Parkin mitophagy and neurodegeneration‐what do we really know in vivo? Curr Opin Genet Dev 2017;44:47–53. 28213158 10.1016/j.gde.2017.01.016

[23] Fritsch LE , Moore ME , Sarraf SA , Pickrell AM . Ubiquitin and receptor‐dependent mitophagy pathways and their implication in neurodegeneration. J Mol Biol 2020;432(8):2510–2524. 31689437 10.1016/j.jmb.2019.10.015 PMC7195237

[24] Cogliati S , Enriquez JA , Scorrano L . Mitochondrial cristae: Where beauty meets functionality. Trends Biochem Sci 2016;41(3):261–273. 26857402 10.1016/j.tibs.2016.01.001

[25] Hirai K , Aliev G , Nunomura A , et al. Mitochondrial abnormalities in Alzheimer's disease. J Neurosci 2001;21(9):3017–3023. 11312286 10.1523/JNEUROSCI.21-09-03017.2001 PMC6762571

[26] Meng H , Yamashita C , Shiba‐Fukushima K , et al. Loss of Parkinson's disease‐associated protein CHCHD2 affects mitochondrial crista structure and destabilizes cytochrome c. Nat Commun 2017;8:15500. 28589937 10.1038/ncomms15500 PMC5467237

[27] Tello C , Darling A , Lupo V , et al. On the complexity of clinical and molecular bases of neurodegeneration with brain iron accumulation. Clin Genet 2018;93(4):731–740. 28542792 10.1111/cge.13057

