# Supplementary material for: Impaired proteasome activity and neurodegeneration with brain iron accumulation in FBXO7 defect
Source: Ann Clin Transl Neurol. 2020 Aug 6;7(8):1436–42. doi: 10.1002/acn3.51095 (PMC7448169; doi:10.1002/acn3.51095)
Supplement: Supplementary file 2 — File S1 . Additional information regarding materials and methods, a supplementary figure and a supplementary table are included in a supplementary material file. Figure S1 . Mitophagy‐related proteins and electron microscopy: (A) Mitophagy markers (PINK1, Parkin, K63‐polyUb, and VDAC) levels analyzed by Western‐blot in mitochondria enriched fractions from patient's and control’s fibroblasts. Error bars represent SEM. n.s.: nonsignificant. (B) Elevated levels of mitochondrial‐specific proteins PINK1 and VDAC1 were detected in mutant cells compared to controls. Error bars represent SEM. Mann–Whitney–Wilcoxon test: *P < 0.05; n.s.: nonsignificant. (C) Analysis of mitochondrial morphology by electron microscopy revealed in ultrathin sections images of mitochondria in control’s and patient’s fibroblasts. Internal scales marker are indicated in the figure. Table S1 . Genetic, clinical, and neuroimaging features of FBXO7 deficiency Supplementary Material [file ACN3-7-1436-s002.docx]

Supplementary material

**Impaired proteasome activity and neurodegeneration with brain iron accumulation in *FBXO7* defect**

**Marta Correa-Vela, MD,^1,2#^ Vincenzo Lupo, PhD,^3,4#^, Marta Montpeyó, MSc,^5^ Paula Sancho, PhD,^3,4^ Anna Marcé-Grau, PhD^1^, Jorge Hernández-Vara, MD^6^, Alejandra Darling, MD, PhD,^7^ Alison Jenkins, MSc,^3^ Sandra Fernández-Rodríguez, MSc,^3^ Cristina Tello, MSc,^3^, Laura Ramírez-Jiménez, MBChB,^8^ Belén Pérez, PhD,^9^ Angel Sánchez-Montáñez, MD,^10^ Alfons Macaya, MD, PhD^1,2^, María J. Sobrido, MD, PhD,^11^ Marta Martinez-Vicente, PhD,^6^ Belén Pérez-Dueñas, MD, PhD,^1,2§*^, Carmen Espinós, PhD,^3,4§*^**

1. Department of Pediatric Neurology, Hospital Universitari Vall d’Hebron, Barcelona, Spain.
2. Universitat Autònoma de Barcelona, Barcelona, Spain
3. Unit of Genetics and Genomics of Neuromuscular and Neurodegenerative Disorders, Centro de Investigación Príncipe Felipe (CIPF), Valencia, Spain.
4. Joint Units INCLIVA & IIS La Fe Rare Diseases, Centro de Investigación Príncipe Felipe (CIPF), Valencia, Spain
5. Neurodegenerative diseases-CIBERNED, Vall d´Hebron, Institut de Recerca, Barcelona, Spain
6. Department of Neurology, Hospital Universitari Vall d’Hebron, Barcelona, Spain.
7. Department of Pediatric Neurology, Hospital Sant Joan de Déu, Barcelona, Spain.
8. Unit of Genomics and Traslational Genetics, Centro de Investigación Príncipe Felipe (CIPF), Valencia, Spain.
9. Department of Molecular Biology, Centro de Biología Molecular Severo-Ochoa UAM-CSIC, Universidad Autónoma de Madrid, Centro de Diagnóstico de Enfermedades Moleculares (CEDEM), CIBER on Rare Diseases (CIBERER), Instituto de Investigación Sanitaria Hospital La Paz (IdiPaz), Madrid, Spain.
10. Department of Pediatric Radiology, Hospital Universitari Vall d’Hebrón, Barcelona, Spain.
11. Neurogenetics Research Group, Instituto de Investigaciones Sanitarias (IDIS), Fundación Pública Galega de Medicina Xenómica, and CIBER on Rare Diseases (CIBERER), Santiago de Compostela, Spain.

**^#§^**These authors have contributed equally.

*Corresponding authors:

Dr. Belén Pérez-Dueñas

Vall d’Hebrón Research Institute (VHIR)

Passeig Vall d’Hebrón 119-129. 08035 Barcelona.

Email: [belen.perez@vhir.org](mailto:belen.perez@vhir.org). Tel: +34 934 89 30 00.

Dr. Carmen Espinós

Centro de Investigación Príncipe Felipe (CIPF)

Unit of Genetics and Genomics of Neuromuscular and Neurodegenerative Disorders

c/ Eduardo Primo Yúfera no. 13. 46012 Valencia. Spain

Email: cespinos@cipf.es. Telf. +34 963 289 680.

**Running head title:** *FBXO7* defect with brain iron accumulation

**Number of characters in the title and running head:** 95 and 45 characters including spaces respectively.

**Number of words in the supplementary file:** word count is up to 2080 words excluding legends and references.

**Number of figures:** 1 supplementary figure and 1 supplementary table.

**Key words (5)**: neurodegeneration with brain iron accumulation; parkinsonian-pyramidal syndrome; *FBXO7* gene; ubiquitin proteasome activity; young onset parkinsonism;

**Relevant conflicts of interests/financial disclosures:** Authors report no disclosures.

**Supplementary information:**

**Methods**

**Genetic studies**

Genetic test was carried out in the proband using a custom gene panel MovDisord-498 (genes included are listed in the Annex) based on SureSelectQXT technology (Agilent Technologies, Santa Clara, CA, USA) for Illumina (San Diego, CA, USA). Data processing from FASTQ file to the Variant Call Format (VCF) files followed Best Practices from Genome Analysis Toolkit (GATK).{Van der Auwera, 2013 #4063} Finally, variant calls were functionally annotated with Variant Effect Predictor (VEP) from Ensembl.{McLaren, 2016 #4064} Variants in homozygosis were prioritized when filtering data, since this family reported consanguinity. Sanger sequencing on an ABI Prism 3130XL analyzer (Applied Biosystems, Foster City, CA, USA) was performed for validation and segregation analysis. To investigate the novelty of the variants, several databases were consulted (1000G, ESP6500, ExAC, gnomAD, ClinVAR, and HGMD® Professional 2020.1). Conservation was analyzed using the GERP and PhyloP software, and pathogenicity was *in silico* predicted with the SIFT, PolyPhen-2 and PROVEAN algorithms.

A read depth approach using gene panel sequencing data was also implemented for the detection of copy number variants (CNVs). Normalized mean coverage (NMC) was divided by the media obtained from all samples’ NMC, and a ratio <0.7 and >1.3 was considered for a loss and gain of exonic targets, respectively.

For the array-CGH, genomic DNA was fragmented and labelled according to manufacturer’s instructions from Agilent Technologies (Genomic DNA Analysis Enzymatic Labeling for Blood, Cells, or Tissues’ protocol Version 7.3 March 2014), and subsequently hybridized with a SurePrint G3 Human High-Resolution Discovery Microarray 1x1M: (AMADID-023642) containing 963331 distinct biological features (Agilent Technologies, Santa Clara, CA, USA). Arrays were scanned in an Agilent Microarray Scanner (Agilent G2565C) according to the manufacturer’s protocol and data extracted using Agilent Feature Extraction Software 12.0.3.2 following the Agilent protocol CGH_1200_Jun14, grid template 023642_D_F_20111015 and the QC Metric Set CGH_QCMT_Jun14. Array-CGH data were analyzed with Cytogenomics 4.0 and interpretation of results were carried out with Cartagenia both software from Agilent Technologies (Santa Clara, CA, USA).

**List of genes included in the gene panel MovDisord-498**

*AARS2,ABCB7,ABCD1,ABHD12,ACAT1,ACO2,ACTB,ADAR,ADCK3,ADCY5,AFG3L2,AHI1,AIFM1,AIMP1,ALDH18A1,ALDH3A2,ALG6,ALS2,AMACR,AMPD2,AMT,ANO10,ANO3,AP4B1,AP4E1,AP4M1,AP4S1,AP5Z1,APTX,ARG1,ARL13B,ARL6IP1,ARSA,ARSI,ARX,ASPA,ASS1,ATCAY,ATL1,ATM,ATN1,ATP13A2,ATP1A2,ATP1A3,ATP2B3,ATP6AP2,ATP7A,ATP7B,ATP8A2,ATR,ATXN1,ATXN10,ATXN2,ATXN3,ATXN7,B4GALNT1,BCAP31,BCKDHA,BCKDHB,BCS1L,BEAN1,BICD2,BSCL2,BTD,C10ORF2,C12ORF65,C19ORF12,C5ORF42,C9ORF72,CA8,CACNA1A,CACNA1B,CACNA1G,CACNB4,CAMTA1,CASK,CC2D2A,CCDC88C,CCT5,CEP290,CEP41,CHCHD2,CHMP1A,CIZ1,CLCN2,CLN5,CLN6,CLP1,COASY,COL18A1,COL6A3,COQ2,COQ4,COQ7,COQ9,COX10,COX15,COX20,COX8A,CP,CPT1C,CSTB,CTDP1,CTSD,CUL4B,CWF19L1,CYP27A1,CYP2U1,CYP7B1,DARS2,DBT,DCAF17,DCLRE1B,DCTN1,DDB2,DDC,DDHD1,DDHD2,DKC1,DLAT,DLD,DNAJC13,DNAJC19,DNAJC6,DNMT1,DYRK1A,EARS2,ECHS1,EEF2,EIF2B1,EIF2B2,EIF2B3,EIF2B4,EIF2B5,EIF4G1,ELOVL4,ELOVL5,EMC1,ENTPD1,EPM2A,ERCC2,ERCC3,ERCC5,ERCC6,ERCC8,ERLIN1,ERLIN2,ETFA,ETFB,ETFDH,ETHE1,EXOSC3,EXOSC8,FA2H,FAAH2,FAM126A,FAM134B,FARS2,FBXL4,FBXO7,FGF14,FLRT1,FLVCR1,FMR1,FOLR1,FOXC1,FOXG1,FOXRED1,FTH1,FTL,FUCA1,FUS,FXN,GAD1,GALC,GAMT,GAN,GATM,GBA,GBA2,GCDH,GCH1,GFAP,GFM1,GFM2,GJB1,GJC2,GLB1,GLDC,GLRX5,GNAL,GNAO1,GOSR2,GPR56,GRID2,GRM1,GTPBP2,GTPBP3,HACE1,HEXA,HEXB,HIBCH,HPCA,HPRT1,HSD17B4,HSPD1,HTRA1,HTRA2,HTT,IARS2,IBA57,IFIH1,IFRD1,INPP5E,ISG15,ITM2B,ITPR1,JPH3,KCNA1,KCNA2,KCNC3,KCND3,KCNJ10,KCTD17,KCTD7,KDM6A,KIAA0196,KIAA0226,KIF1A,KIF1C,KIF5A,KIF7,KLC2,KMT2B,KMT2D,L1CAM,L2HGDH,LAMA1,LIAS,LIPT1,LMNB1,LMNB2,LRPPRC,LRRK2,LYST,MAG,MAN2B1,MARS,MARS2,MCEE,MECP2,MECR,MICU1,MLC1,MMACHC,MMADHC,MME,MPZ,MRE11A,MRPL10,MTFMT,MTHFR,MTPAP,MTTP,MUT,NALCN,NARS2,NDUFA1,NDUFA10,NDUFA11,NDUFA12,NDUFA2,NDUFA4,NDUFA9,NDUFAF2,NDUFAF5,NDUFAF6,NDUFS1,NDUFS2,NDUFS3,NDUFS4,NDUFS7,NDUFS8,NDUFV1,NDUFV2,NHLRC1,NIPA1,NKX2‒1,NOL3,NOP56,NPC1,NPC2,NPHP1,NT5C2,NUP62,OFD1,OPA1,OPA3,OPHN1,PANK2,PARK2,PARK7,PARN,PAX6,PC,PCBD1,PCCA,PCCB,PCNA,PDE10A,PDE8B,PDGFB,PDGFRB,PDHA1,PDHB,PDHX,PDSS1,PDSS2,PDYN,PET100,PEX10,PEX7,PGAP1,PHYH,PIK3R5,PINK1,PITX2,PLA2G6,PLEKHG4,PLP1,PMM2,PMPCA,PNKD,PNKP,PNPLA6,PNPT1,POLG,POLR3A,POLR3B,PPCDC,PPCS,PPP2R2B,PRICKLE1,PRKCG,PRKRA,PRNP,PRPS1,PRRT2,PSEN1,PTEN,PTS,QDPR,RAB39B,RAB3GAP2,RAD1,RARS,RARS2,REEP1,REEP2,RELN,RIPPLY1,RNASEH2A,RNASEH2B,RNASEH2C,RNF170,RNF216,RPGRIP1L,RPIA,RTEL1,RTN2,SACS,SAMD9L,SAMHD1,SCARB2,SCN1A,SCN4A,SCO2,SCP2,SCYL1,SDHA,SDHAF1,SEPSECS,SERAC1,SETX,SGCE,SIL1,SLC16A2,SLC17A5,SLC19A3,SLC1A3,SLC20A2,SLC25A15,SLC25A19,SLC25A42,SLC2A1,SLC30A10,SLC33A1,SLC39A14,SLC46A1,SLC52A2,SLC52A3,SLC6A19,SLC6A3,SLC6A8,SLC9A1,SLC9A6,SMPD1,SNAP25,SNCA,SNX14,SPAST,SPG11,SPG20,SPG21,SPG7,SPR,SPTAN1,SPTBN2,SQSTM1,STUB1,SUCLA2,SUCLG1,SUOX,SURF1,SYNE1,SYNJ1,SYT14,TACO1,TAF1,TARDBP,TBCE,TBP,TCTN1,TCTN2,TCTN3,TDP1,TDP2,TECPR2,TENM4,TERT,TFG,TGFB1,TGM6,TH,THAP1,TIMM8A,TINF2,TK1,TMEM138,TMEM216,TMEM231,TMEM237,TMEM240,TMEM67,TOR1A,TPK1,TPP1,TRAPPC11,TREX1,TRMU,TRPC3,TSEN2,TSEN54,TSFM,TTBK2,TTC19,TTPA,TTR,TUBB4A,TXN2,UBA5,UCHL1,UQCRQ,USP8,VAMP1,VCP,VHL,VLDLR,VPS13A,VPS35,VPS37A,VPS53,VRK1,VWA3B,WDR45,WDR48,WDR73,WDR81,WFS1,WWOX,XK,XPA,XPC,XPR1,ZFR,ZFYVE26,ZFYVE27,ZNF592*

**Cell culture and western-blot**

Skin cultured fibroblasts derived from the patient and controls were grown in high-glucose DMEM medium (4.5 g/L; Thermo Fisher Scientific, Waltham, MA, USA) containing 10% (v/v) fetal bovine serum supplemented with 2 mM glutamine, 100 IU/ml penicillin and 100 mg/ml streptomycin (Invitrogen, Carlsbad, CA, USA).

For western-blot (WB) analysis, fibroblasts were scraped with PBS, centrifuged and cell pellets were lysed with RIPA buffer (50 mM Tris–HCl pH 7.4, 5 mM DTT, 150 mM NaCl, 1% NP-40, 0.5% deoxycholate). Total cell lysates (TCL) protein extracts were quantified using the BCA method (ThermoFisher Scientific, Waltham, Massachussetts, MA, USA); a final amount of 25 μg was employed for WB analysis. For each WB, three independent technical replicates were done. The Mann-Whitney test was used for statistical analysis. Antibodies and their corresponding dilutions are indicated at the end of this section.

**Dihydrolipoamide dehydrogenase assay**

Activity of the DLD (dihydrolipoamide dehydrogenase) enzyme was measured using a modified protocol based on previously reported procedures (Becker DM et al. Clin Chim Acta 1982; 121: 1-9; Chuang et al. Biochem J 1981; 200: 59-67).

**Nonsense-mediated mRNA decay assay**

The effect of nonsense-mediated decay (NMD) mechanism over *FBXO7* transcripts was evaluated by qPCR using fibroblasts from control subjects and from the proband, that were investigated with and without treatment with emetine dihydrochloride hydrate (Sigma-Aldrich, St. Louis, MO, USA), as previously reported.{Kawarai, 2016 #3872} Treated fibroblasts were cultured with 100 μg/mL of emetine dihydrochloride hydrate (Sigma-Aldrich, St. Louis, MO, USA) at 37°C for 6 h before extraction of total RNA. cDNA was obtained with qScript cDNA SuperMix (Quantabio, Beverly, MA, USA) and 0.5 μL were used for each qPCR reaction using PerfecTa SyberGreen Mix (Quantabio, Beverly, MA, USA). The following forward and reverse *FBXO7* primers were used: 5’-AGTCCCTGCTGTGCACCTG-3’ and 5’-CGCTGGAATGTCATCTTGAAGA-3’. Reactions were carried out in LightCycler® 480 System thermocycler (Roche Molecular Diagnostics, Pleasanton, CA, USA). Data was analyzed by Fit Points with LightCycler® 480 SW 1.5 software. *FBXO7* expression levels were normalized to *GAPDH* levels and 2^−ΔΔCT^ method was used for relative quantification. The expression levels of fibroblasts from healthy individual without treatment were assumed as control fibroblasts, and expression levels from the remaining samples were expressed as percentage of the controls. Three independent assays of NMD and three qPCR reactions were done for each assay. A Mann-Whitney-Wilcoxon test was used for analyzing the experimental data.

**Ubiquitin-proteasome system (UPS) activity assay**

Fibroblasts cells were lysed in 10 mM Tris pH 7.8, 1 mM EDTA, 5 mM Mg2Cl, 0.1% Triton X-100. Chymotrypsin-like activity was determined using 10 μg of total cell extracts in a total volume of proteasome activity buffer (10 mM tris pH 7.8, 1 mM EDTA, 0.5 mM DTT, 5 mM Mg_2_Cl, 2 mM ATP), incubating for 60 min at 37 ºC with 0.5 mM of the fluorogenic substrate Z-Leu-Leu-Glu-AMC (Z-LLE-AMC) (Enzo, New York, NY, USA). Fluorescence intensity was quantified by using the Flx800 multi-detection microplate reader (BioTek Instruments Inc., Winooski, VT, USA) with excitation and emission wavelengths at 360 and 460 nm, respectively. Values correspond to six independent samples (from independent wells), each samples was performed in triplicates in the UPS assay and mean value were used.

**Sodium dodecyl sulfate-polyacrylamide gel electrophoresis (SDS-PAGE)**

Fibroblasts cells were collected with phosphate buffered saline 1X (PBS 1X) and centrifuged at 800 g, 4ºC for 5 minutes. The pellet was resuspended in 100 μl of lysis buffer (Tris pH 7.5 20 mM, EDTA 1 mM, NaCl 150 mM, Triton X-100 1%) with protease inhibitors, sonicated and quantified by BCA Protein assay (Thermo Fisher, Waltham, MA, USA). Samples were heated at 95ºC for 5 min after adding loading buffer 6x containing Tris 500 mM, 30% glycerol (Sigma-Aldrich, St. Louis, MO, USA), 10% SDS and 0.6 M DTT. Proteins were resolved by SDS-PAGE on different percentage of polyacrylamide gels, ranging from 8% to 12% and run around 90 min at 120 V in running buffer (25 mM Trizma R Base, 192 mM glycine and 1% SDS). Resolved proteins were transferred onto nitrocellulose membranes during 90 minutes at 200 mA per gel in transfer buffer (25 mM Trizma R Base, 192 mM glycine and 20% methanol) and then blocked with 5% non-fat milk powder in PBS for 1 h at room temperature (RT). Membranes were incubated with the corresponding primary antibodies (indicated at the end of this section) diluted in 4% bovine serum albumin in PBS overnight at 4ºC. Then, we proceeded to the incubation with the corresponding secondary antibodies coupled with horseradish peroxidase and diluted in 5% milk/PBS for 1h at RT. Finally, proteins were visualized using either West Pico SuperSignal Substrate or SuperSignal West Femto (Thermo Fisher, Waltham, MA, USA) on an ImageQuant RT ECL imaging system (GE Healthcare, Chicago, IL, USA). Immunoblots were quantified by densitometry using ImageJ 1.50a.

**Mitophagy-related proteins**

Mitochondrial enriched fractions were isolated from fibroblast with Mitochondrial Isolation Kit for Mammalian Cells (Thermo Fisher, Waltham, MA, USA) according to manufacturer’s instructions. Mitochondrial fraction was resuspended in 100 μl of lysis buffer (Tris pH 7.5 20 mM, EDTA 1 mM, NaCl 150 mM, Triton X-100 1%) with protease inhibitors and quantified with BCA.

Protein markers of mitophagy (PINK1, Parkin, polyUb-K63, VDAC) were immunodetected by WB. Antibodies and their corresponding dilutions are indicated at the end of this section.

**Electronic microscopy**

For electron microscopy studies, wild-type and mutant primary fibroblasts culture were grown in a Lab-Tek Permanox Chamber slide pf 4 wells (Nalge Nunc International, Naperville, IL), washed with fresh 0.1M phosphate buffer (PB) and fixed with 3% glutaraldehyde in 0.1M phosphate buffer (PB) for 2 hours at 37ºC. Subsequently, samples were washed with 0.1M PB, 5 times (5 min. each) and stored at 4ºC in the same buffer. The samples were post-fixed in 2% OsO4 for 1 hour at room temperature and stained in 2% uranyl acetate in the dark for 2 h at 4°C. Then, they were rinsed in distilled water, dehydrated in ethanol and infiltrated overnight in Durcupan resin (Sigma-Aldrich, St. Louis, USA). Following polymerization, embedded cultures were detached from the wells and glued to Durcupan blocks. Finally, ultra-thin sections (0.08 µm) were cut with an Ultracut UC-6 (Leica microsystems, Wetzlar, Germany), stained with lead citrate (Reynolds solution) and examined under a transmission electron microscope FEI Tecnai Spirit BioTwin (ThermoFisher Scientific company, Oregon, USA), using a digital camera Morada (Olympus Soft Image Solutions GmbH, Münster, Germany).

**Antibodies**

| **Antibody** | **Origin** | **Reference** | **Dilution** |
| --- | --- | --- | --- |
| Anti-FBXO7 | Polyclonal mouse | Abnova | 1/1000 |
| Anti-actin^(1)^ | Polyclonal rabbit | Abcam | 1/5000 |
| Anti-actin^(2)^ | Monoclonal mouse | Sigma-Aldrich A5441 | 1/2000 |
| Anti-Parkin | Polyclonal rabbit | Abcam Ab15954 | 1/2000 |
| Anti-VDAC | Polyclonal rabbit | Abcam Ab15895 | 1/2000 |
| PolyUB-K63 | Monoclonal rabbit | Millipore 05-1308 | 1/2000 |
| PINK1 | Polyclonal rabbit | Novusbio BC100-494SS | 1/2000 |
| β-actin | Monoclonal mouse | Sigma-Aldrich A5441 | 1/2000 |

1. Used as protein loading control in WB for detection of FBXO7
2. Used as protein loading control in WBs related to UPS activity assay and mitochondrial studies.

**Supplementary Figure:**

**FIG. S1**. Mitophagy-related proteins and electron microscopy:





1. Mitophagy markers (PINK1, Parkin, K63-polyUb and VDAC) levels analyzed by western-blot in mitochondria enriched fractions from patient's and control´s fibroblasts. Error bars represent SEM. n.s.: non-significant*.*
2. Elevated levels of mitochondrial specific proteins PINK1 and VDAC1 were detected in mutant cells compared to controls. Error bars represent SEM. Mann-Whitney-Wilcoxon test: **P<0.05;* n.s. non significant
3. Analysis of mitochondrial morphology by electron microscopy revealed in ultra-thin sections images of mitochondria in control´s and patient´s fibroblasts.

**Supplementary Table:**

**TABLE S1:** Genetic, clinical and neuroimaging features of FBXO7 deficiency

|  |  |  |  |  | |  |
| --- | --- | --- | --- | --- | --- | --- |
|  | **FBXO7 features** |  | **n** | **%** | |  |
|  |  |  |  |  | |  |
|  | **Gender (n=27)** |  |  |  | |  |
|  |  | Female | 12 | 44,4 | |  |
|  |  | Male | 15 | 55,6 | |  |
|  | **Country (n=27)** |  |  |  | |  |
|  |  | Iran | 10 | 37 | |  |
|  |  | Turkey | 8 | 29,6 | |  |
|  |  | Pakistan | 3 | 11,1 | |  |
|  |  | Italy | 2 | 7,4 | |  |
|  |  | Netherlands | 2 | 7,4 | |  |
|  |  | China | 1 | 3,7 | |  |
|  |  | Morocco | 1 | 3,7 | |  |
|  | **Genetics** |  |  |  | |  |
|  |  | Parental consanguinity | 21/27 | 77,7 | |  |
|  |  | Missense mutation | 14/27 | 51,9 | |  |
|  |  | Nonsense mutation | 13/27 | 48,1 | |  |
|  | Pathogenic reported variants | p.Arg498*: hom | 11/27 | 40,7 | |  |
|  |  | p.Arg378Gly: hom | 10/27 | 37 | |  |
|  |  | p.Leu34Arg: hom | 2/27 | 7,4 | |  |
|  |  | c.1144+1G >T + p.Thr22Met | 2/27 | 7,4 | |  |
|  |  | p.Glu470* + p.Asn51Ser | 1/27 | 3,7 | |  |
|  |  | p.Ser123*: hom | 1/27 | 3,7 | |  |
|  | **Onset features (n=27)** | | | |  |  |
|  | Mean onset age | 23,4 ± 10,2 |  |  | |  |
|  |  | Spasticity | 11/27 | 40,7 | |  |
|  |  | Bradykinesia/Rigidity | 11/27 | 40,7 | |  |
|  |  | Tremor | 3/27 | 11,1 | |  |
|  |  | Walking difficulties | 1/27 | 3,7 | |  |
|  |  | Chorea, tics | 1/27 | 3,7 | |  |
|  | **Motor phenotype (n=27)** | | | |  |  |
|  |  | Parkinsonian-pyramidal | 15 | 55,5 | |  |
|  |  | Spastic paraplegia | 7 | 25,9 | |  |
|  |  | Early onset parkinsonism | 5 | 18,5 | |  |
|  | **Common features (>50% patients reported)** | | | |  |  |
|  | Parkinsonian signs | Postural instability | 15/16 | 93,8 | |  |
|  |  | Bradykinesia | 20/27 | 74 | |  |
|  |  | Rigidity | 20/27 | 74 | |  |
|  | Pyramidal signs | Hyperactive tendon reflexes | 22/27 | 81,5 | |  |
|  |  | Babinski reflex | 21/26 | 80,7 | |  |
|  |  | Spasticity | 19/25 | 76 | |  |
|  | Mixed signs | Dysarthria | 13/15 | 86,7 | |  |
|  | **Less common features (<50% patients reported)** | |  |  | |  |
|  | Parkinsonian signs | Hypomimia | 11/20 | 55 | |  |
|  |  | Monotonous speech | 7/14 | 50 | |  |
|  |  | Resting tremor | 10/27 | 37 | |  |
|  |  | Action tremor | 7/23 | 30,4 | |  |
|  | Oculomotor signs | Reduced upgaze | 7/11 | 63,6 | |  |
|  |  | Slow saccades | 6/10 | 60 | |  |
|  | Extrapiramidal signs | Dystonic features | 10/16 | 62,5 | |  |
|  |  | Myoclonic jerks | 3/3 |  | |  |
|  |  | Chorea | 1/5 |  | |  |
|  |  | Tics | 2/5 |  | |  |
|  |  | Frequent blinking | 5/5 |  | |  |
|  | Other signs | Dysphagia | 10/13 | 76,9 | |  |
|  |  | Urinary incontinence | 3/7 | 42,3 | |  |
|  | **Outcome** |  |  |  | |  |
|  |  | Cognitive decline | 10/26 | 38,5 | |  |
|  |  | Wheelchair bound | 6/16 | 37,5 | |  |
|  |  | Exitus | 3/13 | 23,1 | |  |
|  |  | Bed bound | 2/13 | 15,4 | |  |
|  | **Neuroimaging** |  |  |  | |  |
|  | MRI (n=20) | Normal | 12 | 60 | |  |
|  |  | Cortical atrophy | 6 | 30 | |  |
|  |  | NBIA | 1 |  | |  |
|  | Nuclear imaging (n=4) | 123I-FP-CIT (DaTSCAN) Abnormal | 2 |  | |  |
|  |  | 123I-FP-CIT (DaTSCAN) Normal | 1 |  | |  |
|  |  | 123I-IBZM-SPECT Normal | 1 |  | |  |
|  | **Treatment with levodopa** |  |  |  | |  |
|  |  | LDOPA positive response | 17/18 | 94,4 | |  |
|  |  | LDOPA induced Behavioural problems | 12/13 | 92,3 | |  |
|  |  | LDOPA induced Dyskinesia | 11/13 | 84,6 | |  |
|  |  | LDOPA Motor fluctuations | 7/10 | 70 | |  |
|  |  |  |  |  | |  |

MRI: Magnetic Resonance Imaging; NBIA: Neurodegeneration with brain iron accumulation; 123I-FP-CIT (DaTSCAN): Ioflupane I 123 DaTSCAN; 123I-IBZM-SPECT: I 123 Iodobenzamide Single photon emission computed tomography; LDOPA: levodopa
